# Analysis of complexes formed by small gold nanoparticles in low concentration in cell culture media

**DOI:** 10.1371/journal.pone.0218211

**Published:** 2019-06-14

**Authors:** Stefán B. Gunnarsson, Katja Bernfur, Ulrica Englund-Johansson, Fredrik Johansson, Tommy Cedervall

**Affiliations:** 1 Department of Biochemistry and Structural Biology, Lund University, Lund, Sweden; 2 NanoLund, Lund University, Lund, Sweden; 3 Department of Clinical Sciences in Lund, Lund University, Lund, Sweden; 4 Department of Biology, Lund University, Lund, Sweden; VIT University, INDIA

## Abstract

New nanomaterials are constantly developed with applications in everything from cosmetics to high tech electronics. Assessing their biological impact has been done by analysis of their adsorbed protein corona, *in vitro* cell assays, and larger scale ecotoxicological studies. This has proved to be a huge challenge due to the wide range of available nanomaterials and their unpredictable behaviour in different environments. Furthermore, the enormous number of experimental variables make comparisons difficult. Concentration is one of these variables and can vary greatly depending on the aim of the study. When analysing the protein corona, concentrations are often higher than in cell assays. Using a combination of complementary techniques, we have characterised 20 nm gold nanoparticles in a concentration level commonly used in cell studies. We compare their behaviour in a commonly used, protein rich medium and one protein poor medium over 24 hours. Under these conditions, the NPs were stable in protein rich environment but underwent gradual aggregation in protein poor medium. We characterise the biomolecular corona in both media. In protein poor medium, we can describe the often overlooked aggregation. The aggregates’ morphology is confirmed by cryo-TEM. Finally, in the protein poor medium, by infrared spectroscopy, we have identified the amino acid arginine in the biomolecular corona which drives the aggregation.

## Introduction

The unique properties of nanostructured materials have led to an ever increasing number of applications in various fields, *e*.*g*. electronics, cosmetics, heavy industry, and in pharmaceuticals [1–3]. Nanomaterials are all materials that have at least one dimension in the range of 1–100 nm which gives them properties that are between atomic and bulk. One of the main benefits of nanomaterials is their high surface to volume ratio compared to bulk material, i.e. less material is needed for the same surface activity. Since nanomaterials are defined by their size, they can differ in chemical composition, shape, and electronic and optical properties. Utilization of the enormous potential of nanomaterials calls for unique and highly specific approaches in ensuring their safe application. In biological context, an important property of nanostructures is that their size is the same as many biomolecules, e.g. proteins [4]. While some designed nanomaterials are produced with the aim of *in vivo* use, e.g. in diagnostics and therapeutics, other nanomaterials are intended for applications outside of the human body, e.g. in heavy industry and electronics. For both biological and non-biological designed nanomaterials, it is important to understand the interactions and effect of the material on organisms in the case of both intended and unintended exposure.

Nanoparticles (NPs) are often kept in colloidal stability by electrostatic repulsion between neighboring NPs. The biological environment is a delicate system of biomolecules in dynamic equilibrium. NPs introduced to that system rapidly adsorb biomolecules onto their surface [5], potentially changing the equilibrium. The formation of NP and biomolecule complexes is an important aspect of assessing the impact of NPs on organisms [6, 7]. In addition to proteins, biological fluids contain electrolytes, lipids, amino acids, carbohydrates, and other molecules that might disturb the NPs’ electrostatics and therefore their colloidal stability. The result of mixing these two independently stable systems can therefore lead to formation of various arrangements of NP and biomolecule complexes [8]. These newly formed complexes have a different identity, compared to the naked particle. The physicochemical properties of the complexes need to be characterized as their size will determine if the complex moves by diffusion or sedimentation. This is an important aspect of *in vitro* assays, i.e. how rapidly it reaches the cell [9–12]. The new identity also determines the complexes’ residence time within the organism and the immunological response [13, 14].

Noble metal NPs, e.g. gold and silver, are an attractive option in both diagnostics and drug delivery due to the NPs’ localized surface plasmon resonance (LSPR), detectable by ultraviolet-visible (UV-Vis) spectroscopy [15–20]. The degree of internalization of gold NPs by cells depends on the NPs’ shape [21] and size, where NPs of 50 nm diameter proved to be most readily internalized [22]. Charge also plays a role in cellular interactions with gold NPs, as particles with a positive surface charge were internalized and particles with other charges were not [23, 24]. NP toxicity, assessed *in vitro*, usually entails mixing of a NP of interest with a cell culture medium (CCM) containing blood serum. Proteins from serum can cause a stabilizing effect on the Au NP while serum also provides factors necessary for cell growth [25, 26]. Protein and NP concentration determines the biomolecular corona profile and the probability of NP aggregation which both impact the cells’ response [27, 28]. Albanese and Chen studied the uptake of Au NPs after sodium chloride induced aggregation and coating with transferrin. Their results show that assessment of the role aggregation plays in cell uptake depends on the cell type, mechanism of uptake, and receptor expression [29]. Ha *et al*. recently studied the dosimetry of silver NPs in both an upright and inverted cell culture, to assess the role of diffusion and sedimentation, and found that the particle stability is highly relevant to the effective dose, rather than properties of the bare particle [30].

NP concentration is an often overlooked aspect when studying the behavior of NPs in biological fluids. Concentrations can be reported as molar concentration of Au atoms or NPs, or as Au mass concentration. Concentrations can also vary greatly depending on their aim. Protein corona studies often use high NP concentrations in order to obtain enough material for standard analytical methods. In the literature, concentrations ranging from 0.5 mg mL^-1^ down to approximately 10 μg mL^-1^ are common [28, 31–33]. A review of Au NP toxicological studies shows that concentrations in the nM to μM gold atom concentration range are most common when studying toxicity. In cellular uptake studies it is more common to report molar concentration of NPs and they are usually in the pM range [34]. Evidently, there is a large discrepancy between the concentrations which makes it difficult to draw conclusions from different kinds of studies. In order to make fair and relevant comparison, it is vital to characterize the NPs and their complexes in CCM at conditions similar to those in cell assays.

Our study describes the behavior of 20 nm gold nanoparticles (Au NPs) at the very low particle concentration of 58 pM. This concentration is close to the lower detection limit of the applied analytical methods. Compared to the concentrations described in the previously mentioned review article, the concentration presented here is 14 μM in Au atoms, 58 pM in Au NPs, and 2.8 μg mL^-1^ in mass concentration. The concentration is therefore within the concentration range commonly used in toxicology and cell uptake studies and 2–100 times lower than in protein corona studies. We follow the development of the NPs in two significantly different CCM, one protein rich and one protein poor. The protein rich media chosen was the widely used and well-described CCM RPMI-1640, supplemented with 10% fetal calf serum (FBS) [35]. The protein-poor media chosen was a neural cell culture expansion media, also used in a NP toxicity report by us [36].

## Experimental

### Materials

Gold nanoparticles of 20 and 80 nm diameter (EM.GC20 and EM.GC80), were purchased from BBI Solutions (United Kingdom), RPMI-1640 was purchased from Sigma-Aldrich, DMEM-F12 medium from Invitrogen as described [36], sucrose of proteomics grade was used for gradient preparation purchased from VWR Life Science.

### Sample preparation

We analyzed the interaction of Au NPs with CCM at four different time points over 24 hours, more specifically at 30 minutes, 1 hour, 6 hours, and 24 hours, respectively. We used two different CCM (expansion and RPMI) equilibrated in 5% CO_2_ mixed with Au NPs. The Au NPs were diluted 1:20 in the CCM, which gives final gold concentration of 2.8 μg/mL or 58 pM, and incubated in sterilized Eppendorf tubes until they were analyzed. In supplementary information, the results are compared to 80 nm Au NPs in four concentrations, ranging from 0.18 to 1.8 pM, or 0.57 to 5.7 μg/mL.

### Nanoparticle characterization

All nanoparticle sizes were analyzed by differential centrifugal sedimentation (DCS), dynamic light scattering (DLS), ultraviolet-visible spectroscopy (UV-VIS), and selected samples were further analyzed by SDS-PAGE followed by mass spectrometry and cryo-transmission electron microscopy. DCS was performed using DC24000 disc centrifuge (CPS Instruments, USA) in a linear 8–24% sucrose gradient with a speed of 24,000 RPM. The instrument was set up to continue collecting data until Au NPs of 10 nm diameters reached the detector. In cases where the measuring time was not sufficient to capture all of the NP peak, the run was continued down to 5 nm. The instrument parameters were set to the density of gold, 19.3 g/cm^3^. Dynamic light scattering was performed on DynaPro Plate Reader II (Wyatt Instruments, USA) with acquisition times from 1–4 seconds. The data was analyzed by both cumulant and regularization methods. Absorbance spectra was measured using a ProbeDrum instrument (Probation Labs, Sweden). Absorbance was measured for wavelengths between 240 and 720 nm. For each measurement, a background sample was prepared and subtracted from the Au NP sample. To increase the sensitivity of the absorbance measurement, background was subtracted from each measurement. The background sample was prepared and incubated with the Au NP samples except for one modification. Instead of adding Au NPs to the sample, the equivalent amount of Au NP stock solution supernatant after centrifugation was added, to account for any contribution of the citrate stock solution to the sample pH.

### Mass spectrometry

To obtain sufficient material for SDS-PAGE analysis, 5 mL of NP-media samples were prepared and incubated for 1 hour. After incubation, 1 mL aliquots of the sample were loaded on top of 20% sucrose cushion in five different Eppendorf tubes and centrifuged for 1 hour at 18,000 RPM (31,514 x g) at 4°C (Hettich Mikro 220R, Germany). After centrifugation, the Au NPs had formed a pellet in the bottom of the Eppendorf tube. The supernatant was removed, along with most of the sucrose cushion. The pellet was then removed from the remaining cushion using a 10 μL pipette. The five pellets were pooled together, centrifuged and the sucrose removed. Proteins adsorbed to the Au NPs were then desorbed by boiling in SDS-PAGE sample buffer (60 mM Tris, pH 6.8, 10% SDS, 5% β-mercaptoethanol, 10% glycerol, bromophenol blue) for five minutes. The sample was then loaded onto a 12% polyacrylamide gel in a Mini-PROTEAN Tetra Cell system (Bio-Rad, USA). After electrophoresis, the gel was silver stained using a Pierce Silver Stain for Mass Spectrometry kit. Selected bands were destained and analyzed by mass spectrometry as previously described.[8]

### Cryo-transmission electron microscopy

4 μL of each sample were deposited on a glow-discharged lacey C copper TEM grid, blotted to remove excess liquid, and plunge frozen in liquid ethane. Images at different magnifications were acquired using a JEM-2200FS microscope.

### Attenuated total reflectance Fourier-transform infrared spectroscopy

ATR-FTIR was performed using PerkinElmer Spectrum One FT-IR spectrometer with a Universal ATR accessory. The Au NP samples were centrifuged at 18,000 RPM (31,514 x g) for 30 minutes at 4°C. Then the supernatant was added to the ATR-FTIR crystal, a cap placed over the sample to reduce evaporation and left to equilibrate for 10 minutes at room temperature before scanning as background. Then the supernatant was removed, the pellet added to the crystal and let equilibrate for 10 minutes. Each sample was measured 50–100 times to increase signal to noise ratio.

## Results and discussion

To monitor the interaction of nanoparticles with components of cell culture media (CCM), we incubated gold nanoparticles, with a diameter of 20 nm, in protein rich (RPMI) and protein poor (expansion) media at an Au NP concentration of 2.8 μg/mL. RPMI is supplemented with 10% fetal bovine serum (FBS) (approximately 7 mg/ml proteins) while protein poor CCM only contains proteins in the form of growth factors at concentrations in the ng/mL range. The protein concentration difference in the two media is therefore six orders of magnitude. However, the amino acid, carbohydrate, and salt concentrations are high in both CCM. At low NP concentrations and relatively high concentration of various biomolecules like proteins, carbohydrates, lipids, amino acids, etc. the NP surface will quickly be covered with biomolecules. It is important to consider that NP aggregation that is dependent on direct contact between the surfaces of two Au NPs will happen slower at lower concentration. How long it takes for the system to reach equilibrium will ultimately determine what cells are exposed to and the NPs toxicological impact. Information about the aggregation state and aggregate morphology is crucial when assessing how fast the nanomaterial reaches the cell, if different fractions of the nanomaterial will reach the cell by sedimentation or diffusion, and available surface on the nanostructure for contact with the cell. For example, spherical nanoparticles are more readily taken up by cells than rod-like particles [22]. Upon interaction with biomolecules from the environment, citrate is displaced on the Au NP surface, which may trigger a chain like fractal aggregation [37]. However, a chain of spherical Au NPs would not necessarily behave like the nanorods. The more information we can gather on the system’s behavior, the better we can explain the cell’s response. For a detailed description of the NP behavior, we combine analytical detection methods of different qualities and apply them in a synergistic way.

### Dynamic light scattering

**Fig 1:**
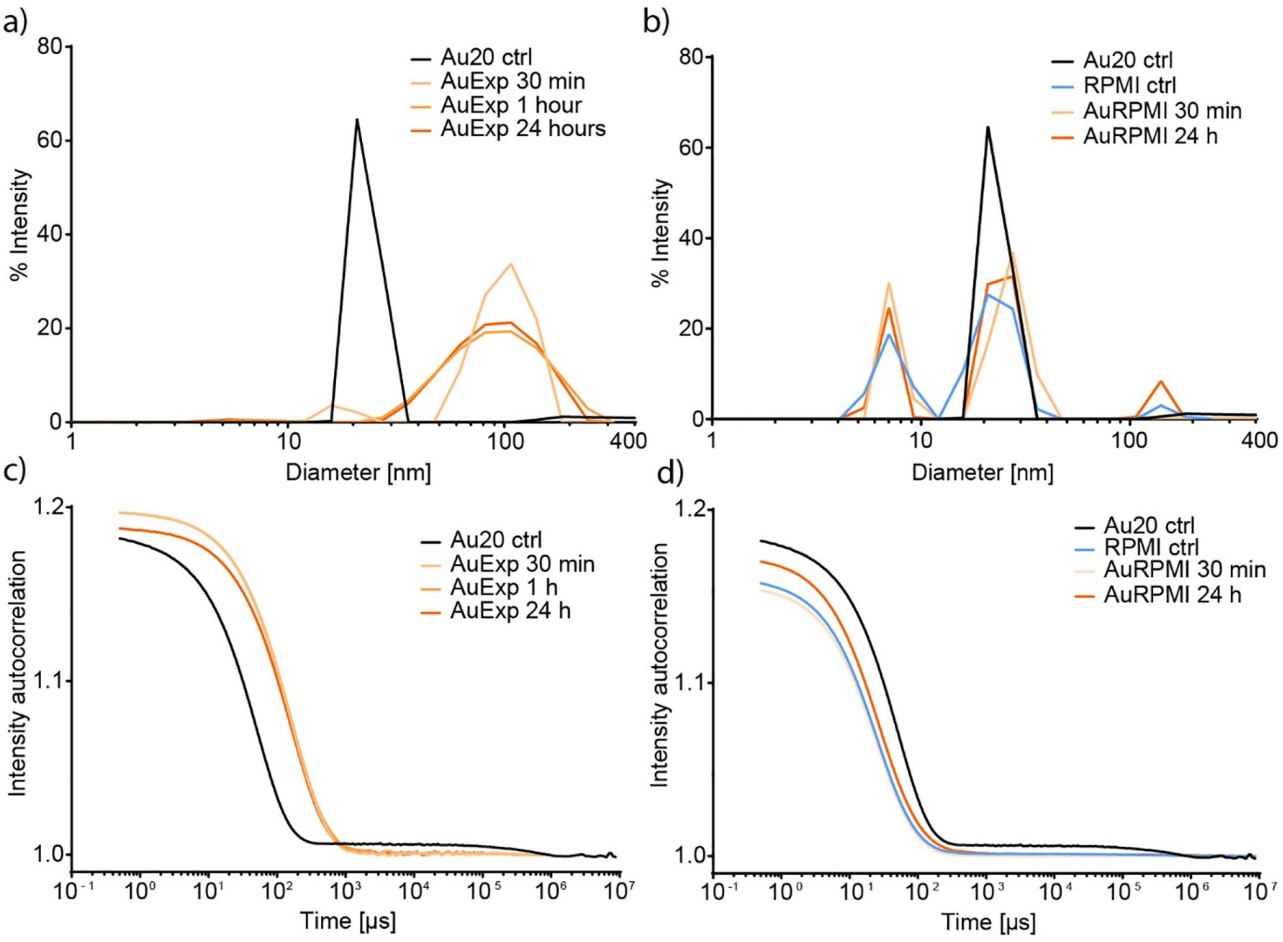
Dynamic light scattering of 20 nm gold nanoparticles in protein poor (expansion) and protein rich (RPMI) cell culture media. a) shows intensity size distribution of 20 nm Au NPs in water (black line), after 30 minutes (light orange), 1 hour (medium orange), and 24 hours (dark orange). b) shows the intensity size distribution of the RPMI media without NPs (light blue), and the size distribution after 30 minutes (light orange), and 24 hours (dark orange). c) shows the intensity autocorrelation as a function of time for the size distribution presented in a), and d) shows the intensity autocorrelation for the size distribution presented in b).

First we determine the NP size distribution over time in protein poor CCM using DLS. DLS is one of the most common methods for NP characterization and obtaining DLS data is often experimentally straightforward. However, the instrument components, calculations and theory is complicated and the interpretation of the DLS data can easily be wrong especially if used by itself.[38] Fig 1a and 1b shows a comparison of the results obtained in protein poor and protein rich CCM, respectively.

#### Protein poor CCM

Fig 1a shows the regularization graph for size intensity distribution of Au20 incubated for 30 minutes, 1 hour and 24 hours after mixing with protein poor CCM. No background size distribution or intensity autocorrelation (Fig 1a and 1c) is shown for protein poor CCM due to the low concentrations of molecules large enough to scatter light. The Au NP control sample (black lines) is the same concentration as in the CCM samples, but diluted in water. Compared to the 20 nm Au NPs in water, larger species are present in the protein poor CCM mixed with NPs. This can be due to aggregation, adsorption of biomolecules from the CCM to the NP surface, or both. Intensity autocorrelation graphs (Fig 1c) rendered that the decay of the function is slower for the NP in protein poor CCM than NP in water, which explains the larger size in the % intensity graph in. 1a. The slope of the decay of the function indicates the size distribution. The slope is less steep after both 1 and 24 hours than 30 minutes incubation, which explains the broadened % intensity peak in Fig 1a. This broadening with peak intensity around a size of 100 nm in diameter can be due to formation of a high variety of similarly sized aggregates, which we will discuss further later. The intensity autocorrelation curves after 1 hour and 24 hours overlap and result in very similar size distributions. Finally, the baseline of the intensity autocorrelation shows some turbulence, presumably due to sedimentation of larger Au NP aggregates. These results indicate that at this low NP concentration, the aggregation process is not very fast.

#### Protein rich CCM

After establishing the NP aggregation behavior in the absence of proteins, we examined the effect of a protein rich environment. Fig 1b shows the % intensity size distribution in protein rich CCM. Supplementing the CCM with 10% of FBS introduces a large variety of biomolecules of different sizes. Due to interference of scattering from the medium, the use of DLS in CCM with high protein concentration and low NP concentration is challenging and the data should be carefully considered before conclusions are drawn. The light blue lines in Fig 1b and 1d shows the background scattering of the CCM. The auto attenuation function in the instrument settings regulates the laser power as to fit with the detector sensitivity. It should therefore be noted that the instrument sets its laser power required to obtain a good signal. For Au NPs in water, 100% of the laser’s capacity was required while only 25% were required for the protein rich CCM without any NPs. The mass concentration of proteins is approximately 2,500 times higher than that of the Au NPs so despite the higher refractive index difference for gold and large size of the NPs, the proteins still dominate the scattering intensity measured. It should also be pointed out that the scattering intensity, according to Rayleigh scattering theory, increases proportionally to the sixth power of the NP’s radius. Most proteins are a few up to tens of nanometers in diameter, which makes it very difficult to distinguish between them and NPs. To support these findings, we measured 80 nm Au NPs at different concentrations, from 0.18 to 1.8 pM (S1 Fig). In 10% FBS, the hydrodynamic diameter was 108 nm, compared to 78 nm for the naked NP, indicating a biomolecular corona of 15 nm. The increased diameter of the NP, and therefore increased scattering intensity, gave a broad peak at the lowest concentration, and only at 1.8 pM (two-fold higher mass concentration than the Au20 used in the experiments), the peak was well distinguishable from the background signal. At lower concentrations the peak was visible but very broad.

The results suggest that the scattering of Au NPs at this low concentration is masked to a large extent by the scattering from the molecules of the CCM, S2 Fig. After adding Au NPs to the CCM, the % intensity distribution is very similar to the CCM itself. The peaks observed around 20 nm are slightly shifted towards larger diameter, from 23.0 nm for RPMI control, to 27.0 and 24.2 nm for 30 minutes and 24 hours, respectively. This increase in diameter of the size distribution can indicate the presence of a higher number of larger species, either Au NP aggregates, NPs with adsorbed biomolecules or complexes of multiple NPs with adsorbed biomolecules. The absolute diameter cannot be derived from the data. Similarly, in the intensity autocorrelation functions, Fig 1d, the decay of the function is similar for RPMI itself and RPMI with Au NPs, 30 minutes after mixing (light orange curve), but the % intensity distribution (Fig 1d) still shows a shift to larger species. After 24 hours, the autocorrelation function decays slower than the RPMI, indicating a shift to larger species. In the intensity size distribution, the average size of the protein and Au NPs are larger than the Au NPs. In contrast no species are observed that are larger than the gold control when examining the auto correlation function. This is likely to be a consequence of the larger uncertainty in the acquired data when proteins are present and the difficulty in deconvolution of the size populations responsible for the scattering. It should further be noted that diluting the particles in water can give different results compared to diluting them in a solvent that has electrostatic properties similar to the CCM, as charged molecules can influence the electric double layer around the particles. Overall, the results from the low concentrations of Au NPs by DLS in a protein rich complex environment should be interpreted as an indication, rather than an absolute value of the size of the NP-biomolecule complex formed.

### Differential centrifugal sedimentation

DLS can only give an indication about the size distribution in the sample. Additional and complementary information of the species formed can be acquired by differential centrifugal sedimentation (DCS). Under high centrifugal force, species of different sedimentation rate pass by the DCS detector that is positioned at a constant length from the center of rotation. The sedimentation rate of a spherical particle at constant centrifugal force and sedimentation height, through a gradient of constant density and viscosity, depends on the particles density and diameter, according to Stokes law S3 Fig. A benefit to DCS, compared to DLS, is that different components of the system are separated during the analysis. However, separating populations of NPs and biomolecules from the environment where they were formed also comes with some considerations, as the populations are no longer in their dynamic, concentration dependent environment.

#### Protein poor CCM

**Fig 2:**
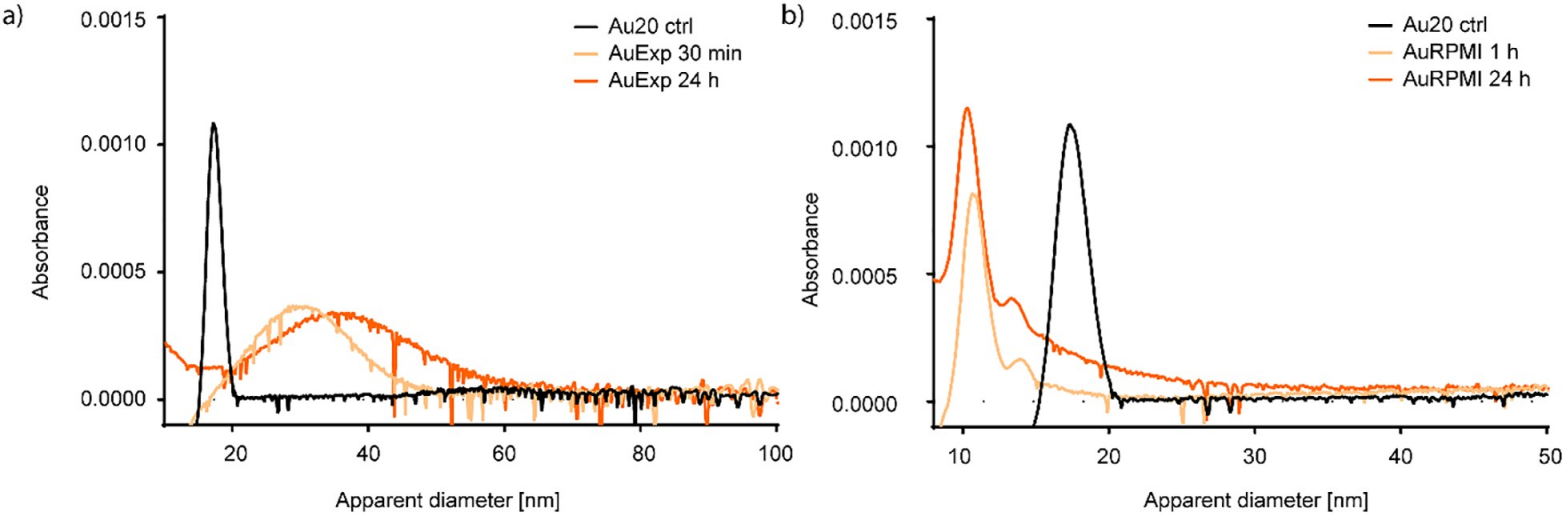
Differential centrifugal sedimentation analysis of the apparent diameter of 20 nm Au NPs in a) expansion and b) RPMI cell culture media.

As in DLS, there are some interpretational difficulties. First of all, the calculations shown as diameter are conversions of sedimentation time according to the program settings. Our data shows the sedimentation profile for spherical particles with the density of gold, 19.3 g/cm^3^. For the control sample, Au NPs in water, this is a fair assumption, however after mixing, we do not know the complex’s density or shape. In case of aggregation and biomolecule adsorption, it is very likely that a variation of shapes is formed, as well as complexes with a wide range of densities. Deviation from a spherical shape will cause delay in sedimentation time, compared to a spherical particle of the same mass, due to higher drag force on the particle. The complex will have an average density of the NPs and biomolecules, according to the number of NPs that make up the complex, and its biomolecular corona thickness, resulting in a lower density than that of gold. Fig 2a (and S4 Fig) shows the sedimentation profile of 20 nm Au NPs in protein poor CCM. Compared to the Au NPs diluted in water (black line), the species formed after 30 minutes (light orange) sediment slower than after 24 hours (dark orange), indicating that under these low Au NP concentration and high concentration of various small biomolecules, equilibrium is not reached immediately. After 30 minutes of incubation, the peak maximum is at 30.6 and 34.6 nm apparent diameter after 30 minutes and 24 hours, respectively. The Au NP control sample diluted in water had an apparent diameter of 17.2 nm. The sedimentation rate does not indicate a single aggregate species. After 30 minutes of incubation the distribution is between 20 and 48 nm, while after 24 hours the distribution is between 20 and 60 nm apparent diameter. These results support the broad distribution obtained by DLS in Fig 1a. The profile also indicates that the system is reaching an equilibrium during the 24 hours, as the apparent diameter is constantly increasing during that time. Despite the low protein concentration, the medium is rich in amino acids that have been shown to induce the formation of fractal aggregates in a less complex environment and will be further discussed later [37].

#### Protein rich CCM

DCS is very useful when studying a system where the interacting components have different densities. Gold has a much higher density than biomolecules, as gold, protein, lipids, and glucose have densities of 19.3, 1.37, 1.06–1.21, and 1.54 g/cm^3^, respectively.[4, 39–41] Using these densities, a 20 nm Au NP with a tightly packed, 10 nm protein corona, would have calculated average density of 3.6 g/cm^3^, and sediment very differently than a 40 nm, spherical aggregate of Au NPs. DLS would, however, show similar results for both complexes. From the sedimentation profiles in Fig 2b and S5 Fig, we see that at high protein concentration, sedimentation of the Au NPs is delayed to smaller apparent diameter. The results indicate that the NPs are stable, with a slight decrease in apparent diameter between 1 to 24 hours of incubation. A smaller apparent diameter can be due to actual smaller size, NP dissolution, decrease in density or deviation from a spherical shape. In our case the most likely scenario is a decrease in average density due to adsorbed proteins on the NP surface, along with some deviation from spherical particles due to the somewhat rough surface of proteins and should not be interpreted as smaller actual size of the NPs [4, 42].

Looking closer at the sedimentation profile of the Au NPs, maximum absorbance is reached at apparent diameter of 10.6 and 10.3 nm after 1 and 24 hours of incubation, respectively. As already mentioned, and shown in S1 Fig, small change in apparent diameter at the smaller sizes translates to quite long sedimentation time. The time difference between 10.6 and 10.3 nm apparent diameter is, under our instrument settings, around 40 seconds. This effect is also very well demonstrated in comparison of the apparent diameters of the species formed under these two conditions. A delay in sedimentation time of 15 seconds for protein poor media gives a difference of 4 nm in apparent diameter. This bias towards the larger diameters should be kept in mind when inspecting the graphical presentation of the data. In protein rich CCM, it is not likely that the size of Au NPs decrease during the reported time span. However, delayed sedimentation time, appearing as decrease in apparent diameter, can have other explanations than a smaller size as deviations from spherical shape or lower density than assumed in the diameter calculations. Consequently, the observed delay in sedimentation of Au NPs in protein rich CCM is either because of a change in shape and/or density. The later explanation is plausible as we know that the Au NPs bind proteins and lipids which have much lower density than gold.

### Ultraviolet-visible spectroscopy

Au NPs in CCM exhibit different characteristics in DLS and DCS. In protein poor CCM, both methods clearly detect formation of larger aggregates. However, the apparent diameter differs, as shown in Figs 1a and 2a, which could be due to aggregate shape or composition. In protein rich media, no significant change can be observed by DLS but Au NPs clearly exhibit slower sedimentation rates, probably due to changes in the density after proteins and lipids bind to the particle surface. To distinguish between different alternative explanations, a third characterization method, ultraviolet-visible spectroscopy, was applied.

#### Protein poor and protein rich CCM

**Fig 3:**
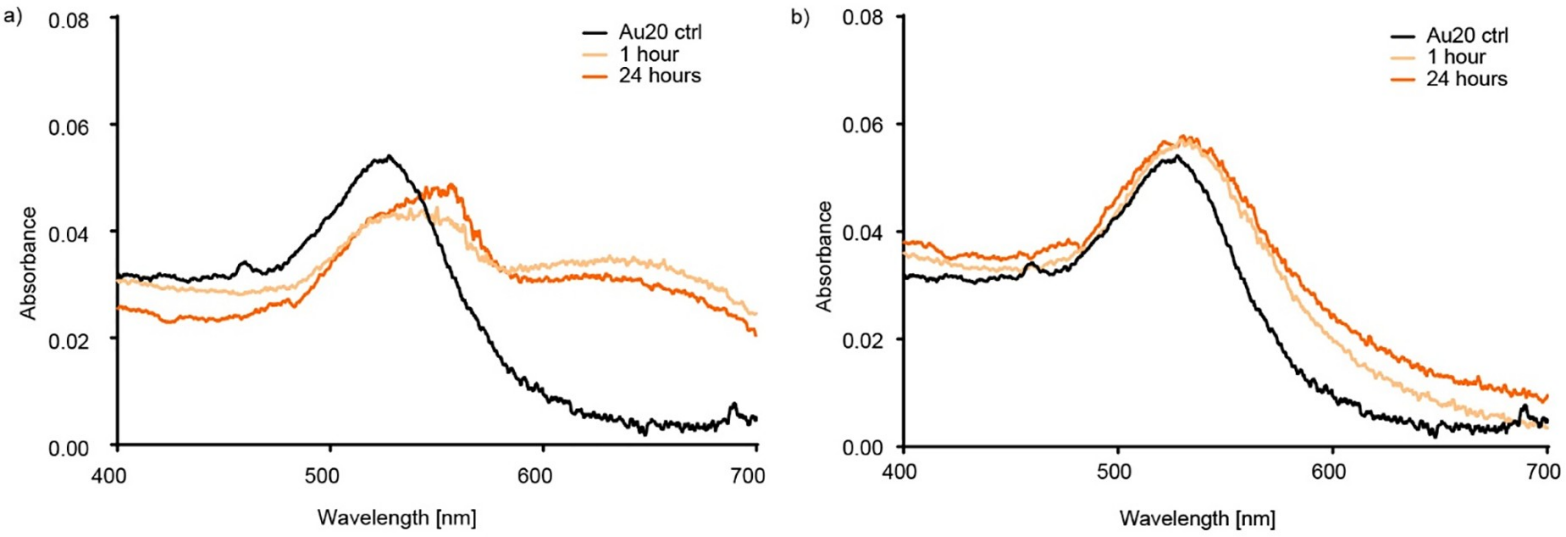
Ultraviolet-visible spectroscopy of Au20 in a) expansion and b) RPMI CCM.

Fig 3 shows the absorbance spectra for 20 nm Au NPs in protein poor and rich CCM, respectively. The absorbance spectra both show a shift to higher wavelength in their maximum absorbance. Due to the differences in CCM compositions, the explanations for this are probably different. Fig 3a shows, for Au NPs in protein poor CCM, a shift in absorbance from 524 nm for the Au NP in water to a broad peak between 535 and 560 nm, as well as a broad peak around 650 nm. Both these peaks have been attributed to aggregated Au NPs [15]. The shift at lower wavelengths is attributed to spherical aggregates and the new peak around 650 nm to fractal aggregates; irregularly shaped aggregates that in our system remain in suspension for long enough to be measured by UV-VIS spectroscopy [37]. In protein rich medium, there is only a shift in the absorbance peak at lower wavelength during the incubation time. After 1 hour of incubation in the protein rich medium, the maximum absorbance had shifted from 524 nm (for Au20 control) to 534 nm and the shift is stable over the 24 hour incubation time. This shift is likely due to the binding of biomolecules to the gold surface changing the electron field and thereby the absorbance maximum [43, 44]. Pollitt *et al*. presented equations to assess the thickness of gold NP coatings [45]. Assuming a RI of 1.45 for the protein layer, the observed shift in maximum absorbance wavelength translates to a protein layer 12–13 nm in thickness [45].

Noble metal nanoparticles have a unique property for detection of changes in their surface environment, due to oscillation of their conduction band electrons [17, 46]. The relatively easily detected shift in absorbance spectra upon surface adsorption has made gold and silver nanoparticles an attractive option in diagnostic tools [19]. Similarly, this unique property can be used to study the adsorption of biomolecules in a complex biological environment. Upon adsorption of biomolecules to the NP surface, the thickness of the adsorbed layer is proportional to the shift in the absorbance peak [43, 44]. Similar to DLS, the method is non-invasive and analyzes the sample without any manipulation or pre-treatment. Interpretation of the data can, however, become troublesome during aggregation, especially when sedimenting aggregates are formed and scattering can cause background absorbance. The absorbance spectra can also give information about the aggregate morphology. Depending on the shape of the aggregate, different absorbance effects are observed. An increase in absorbance between 650 and 700 nm indicates the formation of chain shaped aggregates while spherical aggregates behave like a particle of higher diameter with a smaller shift to a higher wavelength. Chegel et al. used a model of gold NP aggregation induced by small thiol molecules and fitted to experimental results. The absorbance spectra indicated a distribution of monomer- and dimer, linear trimer and hexamer and a spherical 13-mer.[15]

### Cryo-TEM

**Fig 4:**
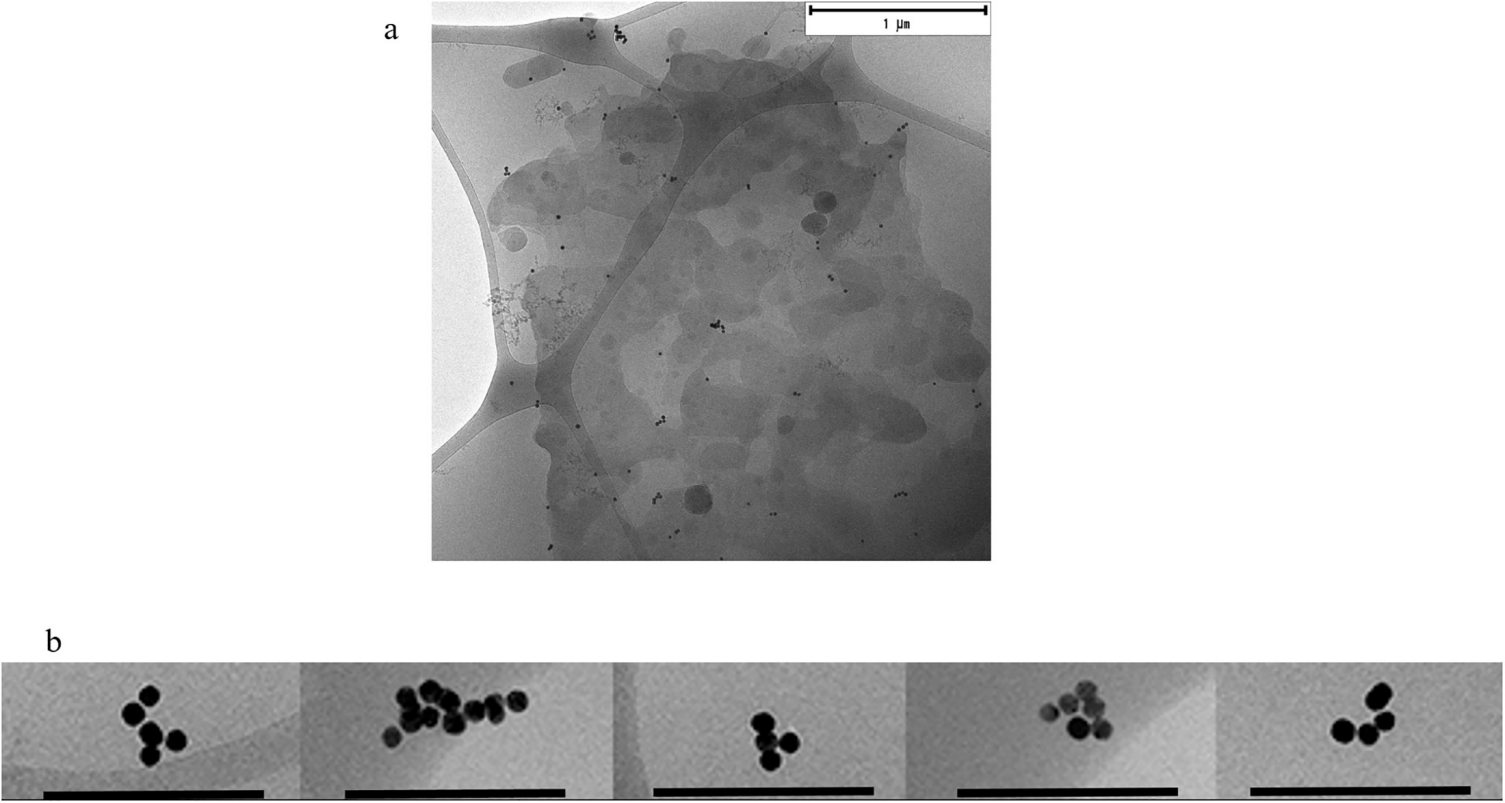
Cryo-TEM of 20 nm Au NPs (a) after 1 hour and (b) after 24 hours of incubation in expansion CCM (scale bar 200 nm).

A fourth characterization method, cryo-transmission electron microscopy (cryo-TEM), was applied to confirm the fractal aggregation in protein poor CCM. Cryo-TEM is an extremely valuable tool when studying biological systems. By flash-freezing the sample of interest, an almost real-life image of the sample can be obtained. In a system of biological fluids and aggregation-prone NPs, it is very useful to look at the sample in a hydrated state, as opposed to regular transmission electron microscopy where the sample needs to be dried to withstand the vacuum conditions when the image is produced. This drying process can easily cause artifacts in the imaged aggregate. Fig 4 shows a distribution of Au NP species, aggregation states, aggregate morphologies and also gives an idea about how far apart these species are while the system approaches equilibrium. The observed aggregate morphology confirms the conclusions drawn by the first three methods. No aggregates could be described as spherical, and populations of Au NP monomers, dimers, linear trimers, random 11-mers and many structures in between can be seen after 1 hour of incubation. Interestingly, after 24 hours of incubation very few monomers were found but more of non-spherical aggregates shown in Fig 4b to 4f. Cryo-TEM is an expensive and low throughput technique but it is invaluable as a confirmation the type of characterization presented in this study. Due to the low throughput of the method, relatively small sample volume and low concentration of NPs, S6 Fig, it is very likely that monomers of Au NPs still remain in suspension, S7 Fig. For a homogenous colloidal system, altering the stability e.g. by adding electrolytes or changing the pH, repulsive forces can be overcome by attractive forces which causes NP aggregation. Depending on the difference in repulsive electrostatic and attractive van der Waals forces [35], the aggregation can be described as diffusion limited colloid aggregation (DLCA) or reaction limited colloid aggregation (RLCA) [47, 48]. The shape of an aggregate will dictate its settling velocity under centrifugation in DCS. Adsorption of small molecules to NP surface has been shown to affect the aggregation rate and mechanism, while amino acids can be classified by how readily they induce fractal aggregation. Doyen *et al*. found that arginine and histidine induced fractal aggregate formation most efficiently. However, their study did not assess the aggregation process in the presence of more than one amino acid. Their results show that positively charged amino acid side chains interact with the gold surface directly, and displace the citrate molecules [37]. Furthermore, free thiol groups on various chemical species are often used to form self-assembled monolayers (SAMs), as a covalent Au-S bond is formed. Protein poor CCM contains both cysteine, the free form of the amino acid, as well as cystine, the oxidized dimer of cysteine.

The radius of fractal aggregates can be described by the following equation [49, 50]

$$i\;=\;k_{f}{\left(\frac{R}{R_{p}}\right)}^{D_{f}}$$

Where i is the number of particles in the aggregate, k_f_ is a prefactor dependent on the aggregate, R is the aggregate radius, R_p_ the radius of primary particles, and D_f_ is the fractal dimension. Using the radius obtained by DLS (15–100 nm), k_f_ of 1.3, and D_f_ between 1.8 and 2.1, the aggregates in Fig 4 should have a diameter of 50–110 nm, with 3 < i < 15 and fits well with the diameter estimated by DLS.

### Biomolecular corona in protein rich CCM

Above we have described the NPs’ characteristics, including their surface properties and aggregation state. As the aggregation state is determined by the surface chemistry, we will turn our attention to the biomolecular corona. Previous studies have mainly focused on the protein corona, but the corona also contains lipids [8, 51, 52] and likely carbohydrates. At low NP concentration levels, determining the biomolecular corona is a challenge. However, by combining the pellets, formed in RPMI supplemented with 10% FBS, from 5 experiments, sufficient amount of material could be obtained for analysis by SDS-PAGE, S8 Fig, and mass spectrometry (see materials and methods for details).

We identified several proteins (S1 Table) in the Au NPs’ corona, of which many have been previously described in the literature [31]. Proteins of a very broad size range are found on the NP surface or incorporated in complexes with the NP and other proteins. Alpha-2-macroglobulin is the largest protein found in the corona. It has a molecular weight of 725 kDa and its dimensions are approximately 10 nm in diameter and 15–20 nm in length [53]. The protein is therefore of similar size to the smaller Au NPs and will considerably change the size and density of the NP protein complex. The lower density slows down the sedimentation rate, resulting in a smaller apparent diameter of the complex by DCS. Lipid binding proteins as apolipoprotein E and gelsolin were also found in the corona. It has been shown for polymeric and TiO_2_ NPs that the lipids remain on the NP surface after binding and even have a preferential binding compared to other proteins [8, 51, 52]. Bound lipids will significantly change the complex’ density. Apolipoprotein E is part of the lipoprotein particle chylomicrons which transport lipids in blood from the intestine. The density of chylomicrons is less than 1 g/cm^3^ and their size can be more than a micrometer. The low NP concentration made it impossible to determine the lipid concentration in the pellet.

### Biomolecular corona in protein poor CCM

The same procedure used in protein rich CCM failed to extract proteins from the complex formed in protein poor CCM. This was not surprising since the only proteins in the CCM are growth factors in the ng/mL range. However, the Au NPs aggregate in protein poor CCM after dilution but not in pure water indicating a change on the NP surface, Figs 1a and 2a. Protein poor CCM is still rich of amino acids, carbohydrates, vitamins, and ions. In order to gain information about the Au NP’s surface after mixing with protein poor CCM, attenuated total reflection Fourier-transform infrared spectroscopy (ATR-FTIR) was applied. It is a very powerful method for detecting the presence of chemical species. By studying the absorbance of infrared radiation by the vibrations of chemical bonds, we can locate characteristic absorbance wavelengths for different chemical bonds.

**Fig 5:**
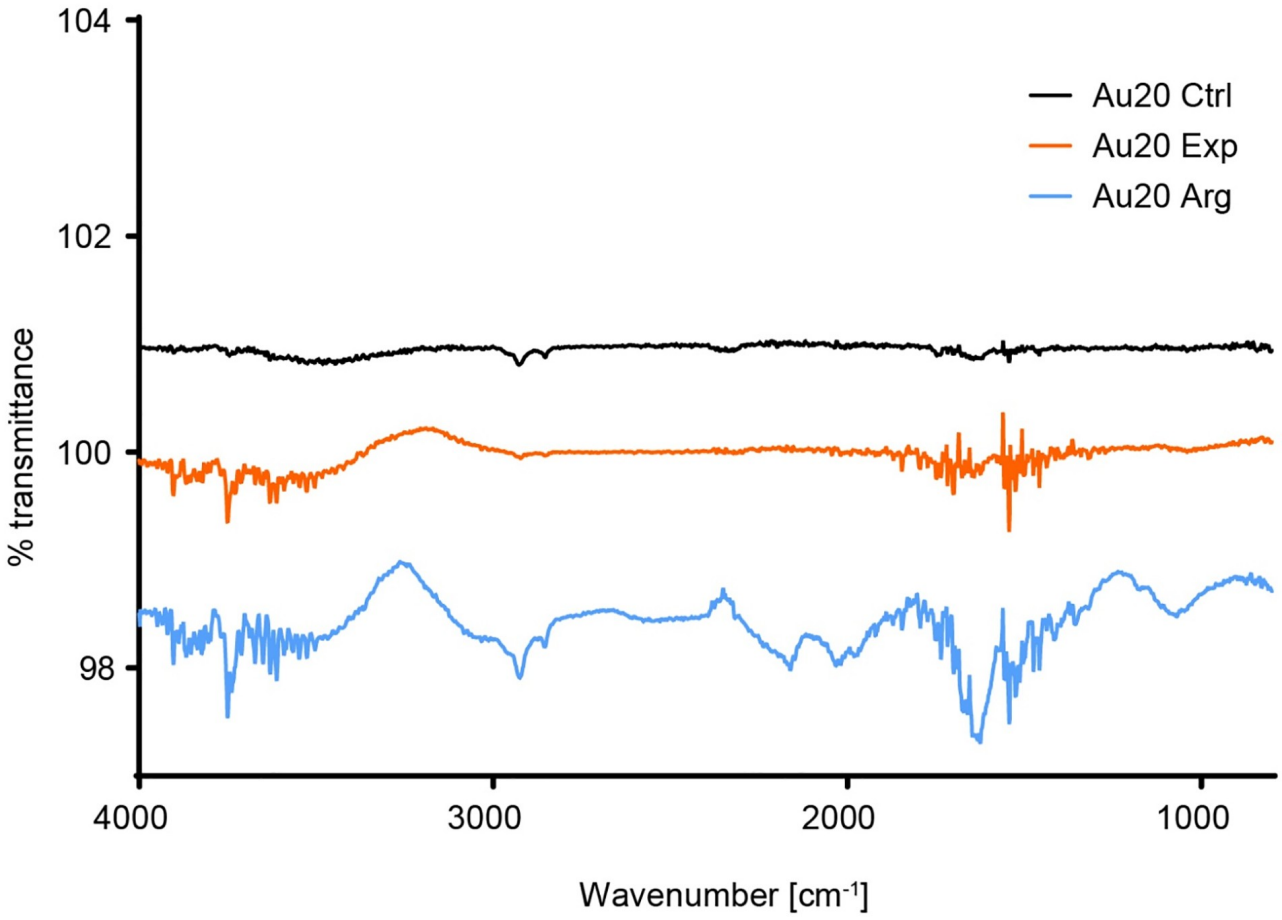
Attenuated total reflection Fourier-transform infrared spectroscopy of Au20 from stock solution (black), after 24 hours of incubation in expansion CCM (orange), and 24 hours incubation in 0.7 mM arginine (blue).

There are some characteristic peaks for the chemical species present in the CCM that adsorb to the Au NP surface, Fig 5. At 1,546 cm^-1^, N-H bend is observed, at 2,924 and 2,852 cm^-1^ C-H stretches, 1,673 and 1,633 cm^-1^ are characteristic for arginine sidechains[54] and the multiple peaks between 1,797 and 1,718 cm^-1^ are due to C = O stretch. For comparison, Au NPs were incubated for 24 hours in 0.7 mM arginine and the pellet analyzed. Clear similarities can be seen between the two samples. However, handling the larger aggregates formed in arginine made it easier to apply the pellet to the crystal, compared to the pellet formed in protein poor CCM. The formation of fractal aggregates, previously shown to be induced by various amino acids in isolation, and the characteristic signal of arginine in ATR-FTIR, show that even if there is not a protein corona on NPs, there is still a biomolecular corona and it drives the aggregation.

### Summary of methodological approach and results

**Fig 6:**
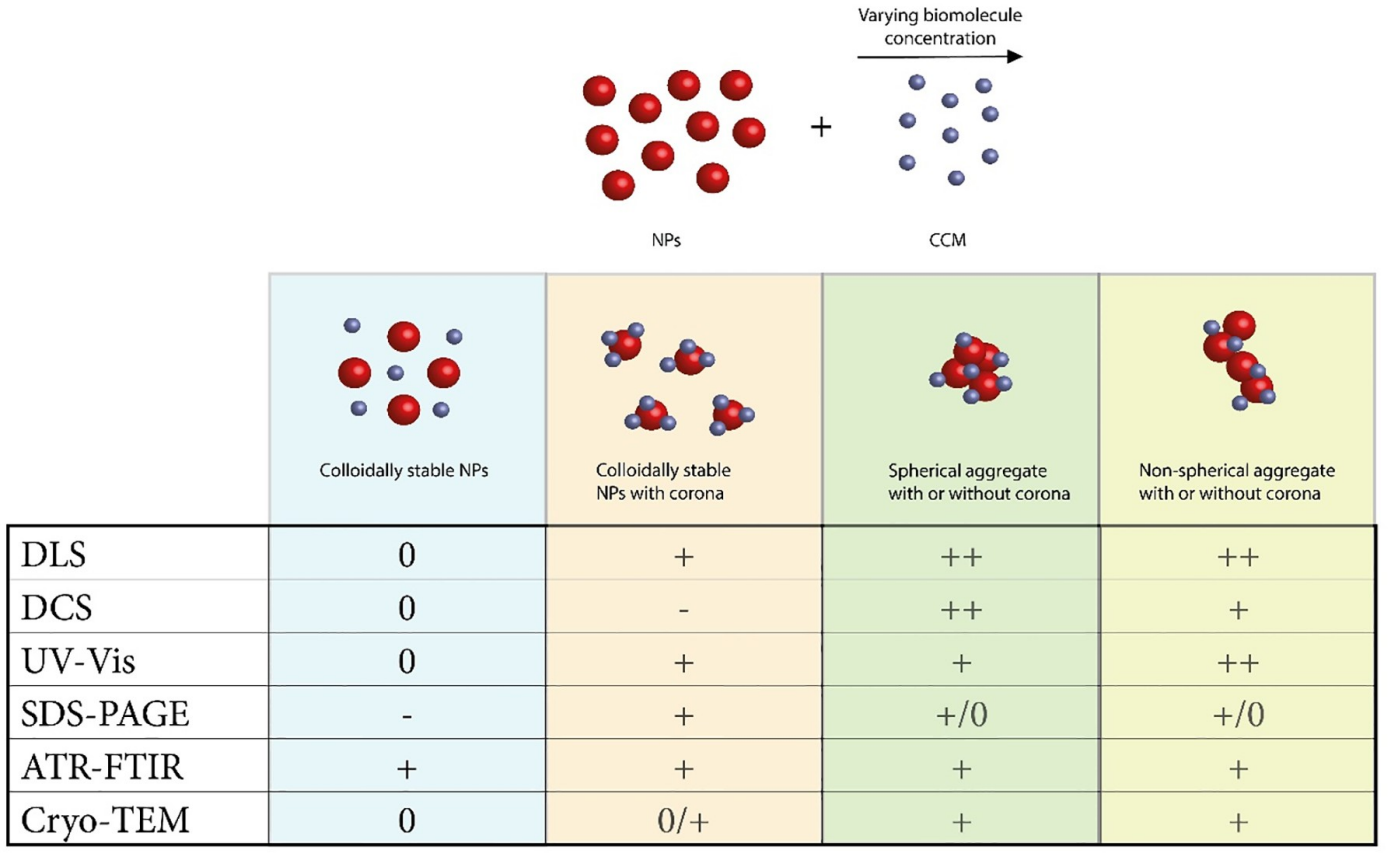
A summary of information gained from different analysis methods. Due to the varying concentrations of proteins (yellow) and other biomolecules (blue spheres) in different CCM, there are in general four different results after mixing. Colloidally stable NPs, NPs with protein and biomolecular corona that remain stable in suspension, spherical aggregates with or without corona, and non-spherical aggregates with or without a corona. The table explains how different detection methods give information about the events. DLS shows hydrodynamic diameter changes, DCS shows faster (+) or slower (-) sedimentation depending on the complexes formed, and UV-Vis shows small shift (+) or a new peak (++) around 650 nm. SDS-PAGE is applicable for all samples, however if no increase in size compared to naked particles, no proteins are likely to be found. ATR-FTIR can give information for all samples about the chemical composition of the sample. Cryo-TEM will not give too much further information about stable systems, but is very informative for aggregated species. All methods require thorough background measurements to distinguish between NPs and CCM.

The benefits of each methodological approach of the study is summarized, Fig 6. The first scenario, a colloidally stable NP without an adsorbed corona is not likely and can be measured by ATR-FTIR to confirm that there are not any adsorbed, smaller species on its surface. The second scenario of colloidally stable NPS with an adsorbed corona is often the result of mixing NPs with a large excess of proteins. The increased hydrodynamic diameter by DLS could indicate either aggregation or adsorption. Slower sedimentation by DCS, however, could only be due to corona formation. Its corona can be studied by SDS-PAGE and ATR-FTIR, while cryo-TEM will not add much. In order to distinguish between scenarios two and three, the main difference is noticed in DCS as spherical aggregates sediment rapidly. Cryo-TEM is very beneficial in this scenario to confirm the aggregate morphology. The fourth scenario, non-spherical aggregation, will result in a slightly slower sedimentation than for spherical aggregates and a difference in their UV-Vis spectra as the peak at 650 nm will be more prominent for the non-spherical aggregates.

## Conclusions

In protein poor CCM, a slow development of fractal aggregation was observed. The first indication of the aggregate morphology was observed when comparing the sedimentation and diffusion behavior of the aggregates by DCS and DLS (Figs 1a and 2a), respectively. Slow sedimentation of the aggregates indicated non-spherical morphology. The appearance of two peaks in the absorbance spectra, around 540 and 650 nm supports our conclusion and cryo-TEM pictures confirm the aggregate morphology. Finally, ATR-FTIR confirms the presence of biomolecules known to induce fractal aggregation on the NP surface. In protein rich CCM, the NPs were stable towards aggregation. The high relative concentration of proteins compared to the NPs made it impossible to measure their actual hydrodynamic diameter by DLS as we could only detect a shift to larger species in the average size distribution. Combining DLS and DCS, it is possible to rule out aggregation as the cause of this increase in size, since the smaller apparent diameter is caused by the adsorption of relatively low density biomolecules to the high density Au NPs. The UV-Vis absorbance spectrum supports this conclusion since there is only one stable peak observed at 534 nm. Characterizing the complexes formed when Au NPs in a very low concentration interact with biomolecules is a key step in assessing their biological impact. In order to study the development of the biomolecular corona in concentrations similar to those used in cell studies, we needed to account for the low concentration with a combination of analytical methods. Doing so we reveal information that no single method can.

## Supporting information

S1 Fig Dynamic light scattering analysis of 80 nm Au NPs. (DOCX)

S2 Fig Dynamic light scattering of Au20 NPs in RPMI after 1 hour of incubation. (DOCX)

S3 Fig Apparent diameter as a function of time for the conditions used in this study. (DOCX)

S4 Fig DCS analysis of Au20 NPs in protein poor CCM after 30 minutes and 24 hours. (DOCX)

S5 Fig DCS analysis of Au20 NPs in protein rich CCM over 24 hours. (DOCX)

S6 Fig Cryo-TEM of 20 nm Au NPs diluted to the same concentration as used in the experiment. (DOCX)

S7 Fig A single Au20 NP after 24 hours incubation in protein poor CCM can be seen on the bottom part of the picture. (DOCX)

S8 Fig SDS-PAGE gel of proteins extracted from protein corona of Au NPs after 1 hour incubation in protein rich CCM. (DOCX)

S1 Table Identified proteins in the corona. (DOCX)
