# Supplementary material for: Analysis of complexes formed by small gold nanoparticles in low concentration in cell culture media
Source: PLoS One. 2019 Jun 14;14(6):e0218211. doi: 10.1371/journal.pone.0218211 (PMC6568402; doi:10.1371/journal.pone.0218211)
Supplement: S1 Fig — (DOCX) [file pone.0218211.s001.docx]

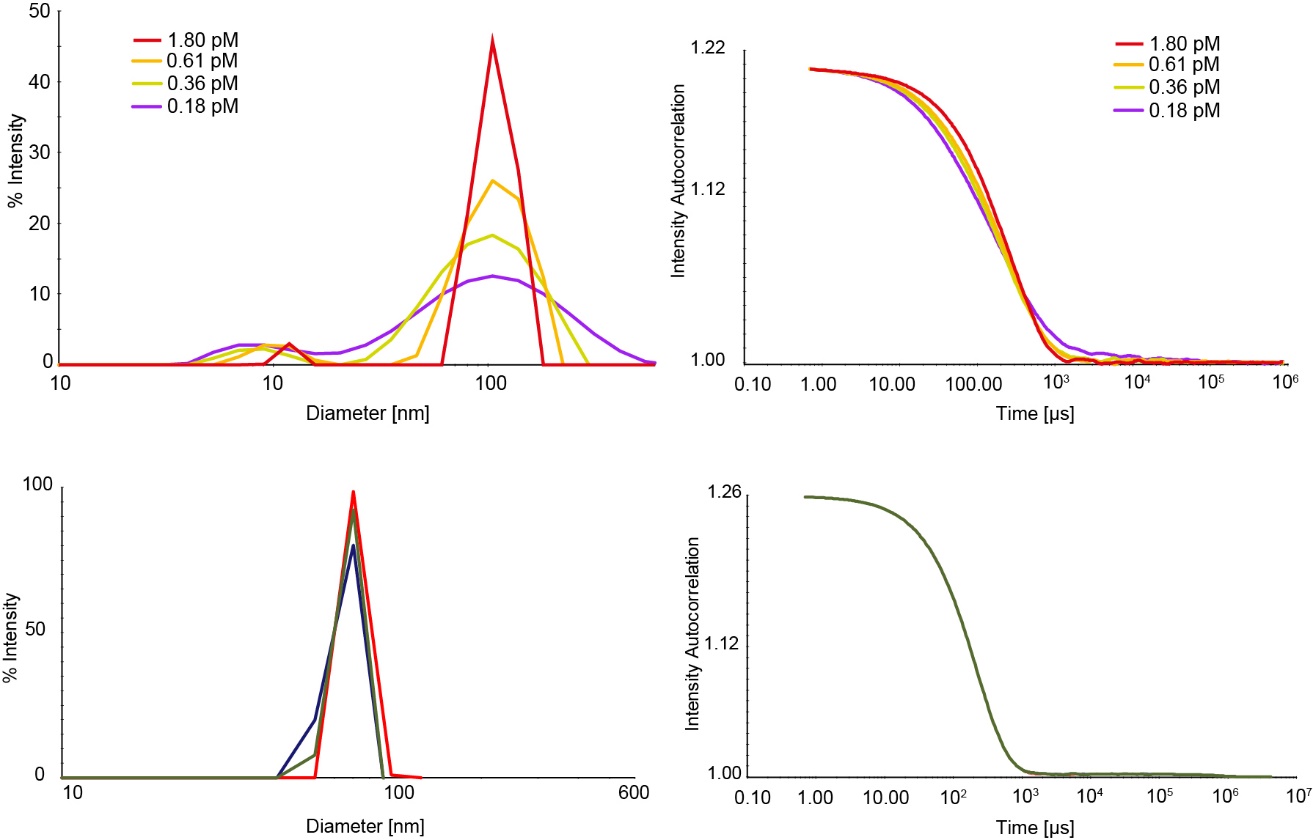


b)

a)

d)

c)

**S1 Fig.** Dynamic light scattering analysis of 80 nm Au NPs. Different concentrations of Au80 in 10% fetal calf serum, a) intensity size distribution. As Au80 concentration is increased, light is scattered by gold rather than the protein of the FCS. b) shows the intensity autocorrelation, used to obtain a), the highest Au80 concentration (red line) has the longest decay time and the steepest slope. This results in a sharper peak, with hydrodynamic diameter of 108±8 nm. Figure c) shows Au80 diluted in water, and d) its corresponding intensity autocorrelation graph. The hydrodynamic diameter of the NPs in water is 78±2 nm.
