# Supplementary material for: Analysis of complexes formed by small gold nanoparticles in low concentration in cell culture media
Source: PLoS One. 2019 Jun 14;14(6):e0218211. doi: 10.1371/journal.pone.0218211 (PMC6568402; doi:10.1371/journal.pone.0218211)
Supplement: S2 Fig — (DOCX) [file pone.0218211.s002.docx]

**Differential centrifugal sedimentation**


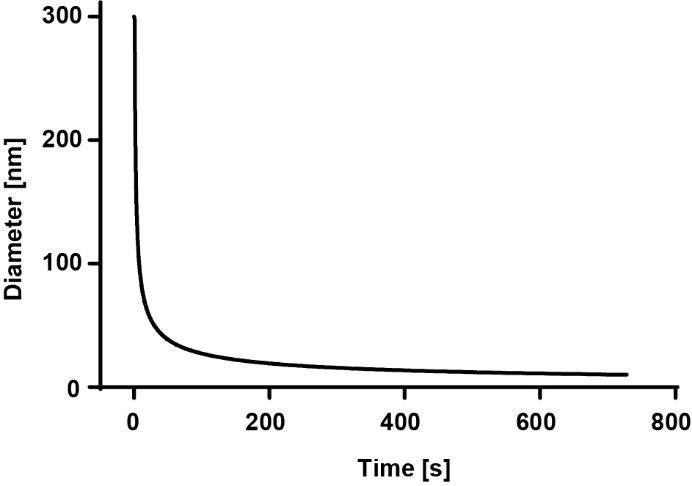


**S3 Fig.** Apparent diameter as a function of time for the conditions used in this study. According to Stoke’s law, the diameter is proportional to the square root of the inverse of the sedimentation time. This effect is clearly shown in fig. S1, where the diameter as a function of time is shown. In the first 60 seconds, particles of sizes from 300 nm to 35 nm pass by the detector, while it takes 728 seconds for 10 nm particles to sediment the same distance.
