# Supplementary material for: Analysis of complexes formed by small gold nanoparticles in low concentration in cell culture media
Source: PLoS One. 2019 Jun 14;14(6):e0218211. doi: 10.1371/journal.pone.0218211 (PMC6568402; doi:10.1371/journal.pone.0218211)
Supplement: S4 Fig — (DOCX) [file pone.0218211.s004.docx]

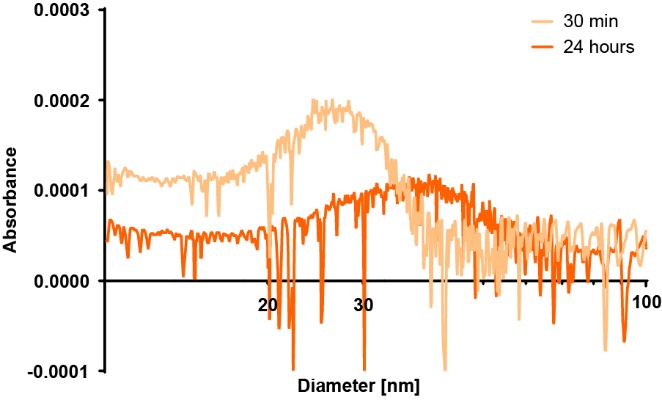


**S4 Fig.** DCS analysis of Au20 NPs in protein poor CCM after 30 minutes and 24 hours A technical detail, important for the presentation of the DCS data, is that when multiple samples are injected into the centrifuge disc, constantly longer time is needed to sediment samples of the same apparent diameter. Therefore, showing the sedimentation time can be misleading and for appropriate comparison between samples, a conversion is necessary. This conversion is referred to here as apparent diameter, i.e. assuming that the sample measured is of the same density as pure Au NPs and spherical. On the y-axis is absorbance, i.e. all the light that does not reach the detector both through absorbance and scattering of the sample.
