# Supplementary figures and images for: Analysis of complexes formed by small gold nanoparticles in low concentration in cell culture media
Source: PLoS One. 2019 Jun 14;14(6):e0218211. doi: 10.1371/journal.pone.0218211 (PMC6568402; doi:10.1371/journal.pone.0218211)

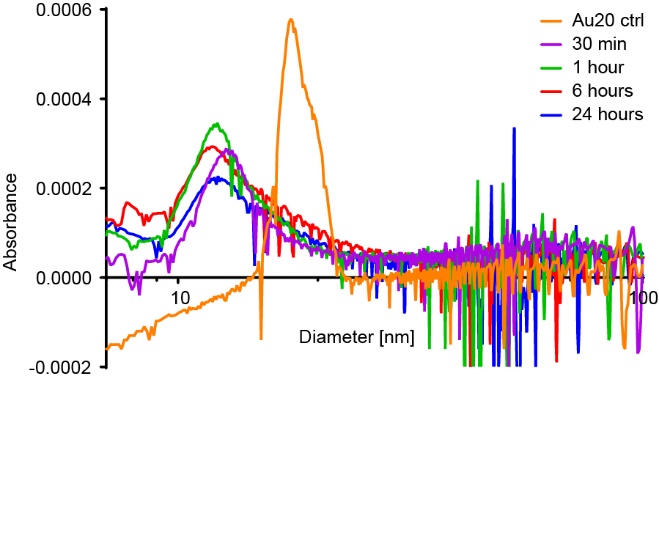


**S5 Fig.** DCS analysis of Au20 NPs in protein rich CCM over 24 hours.

Supplement: S5 Fig — (DOCX) [file pone.0218211.s005.docx]

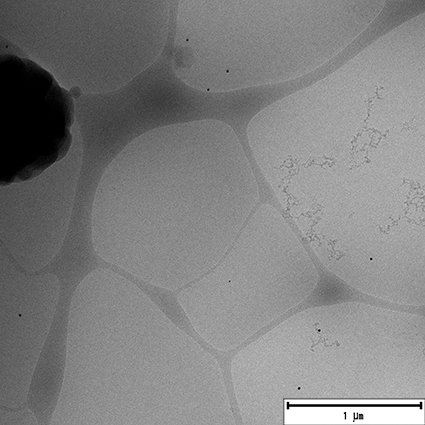


**S6 Fig**. Cryo-TEM of 20 nm Au NPs diluted to the same concentration as used in the experiment.

Supplement: S6 Fig — (DOCX) [file pone.0218211.s006.docx]
