# Supplementary material for: Analysis of complexes formed by small gold nanoparticles in low concentration in cell culture media
Source: PLoS One. 2019 Jun 14;14(6):e0218211. doi: 10.1371/journal.pone.0218211 (PMC6568402; doi:10.1371/journal.pone.0218211)
Supplement: S7 Fig — (DOCX) [file pone.0218211.s007.docx]

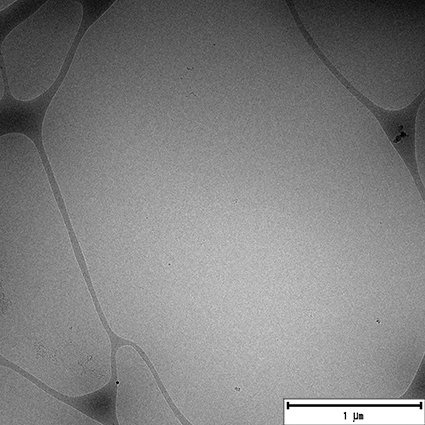


**S7 Fig.** A single Au20 NP after 24 hours incubation in protein poor CCM can be seen on the bottom part of the picture.
