# Supplementary material for: Analysis of complexes formed by small gold nanoparticles in low concentration in cell culture media
Source: PLoS One. 2019 Jun 14;14(6):e0218211. doi: 10.1371/journal.pone.0218211 (PMC6568402; doi:10.1371/journal.pone.0218211)
Supplement: S8 Fig — (DOCX) [file pone.0218211.s008.docx]

**Mass spectrometry**


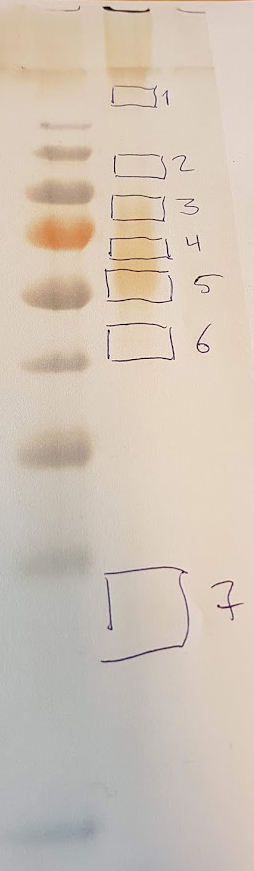


170
130
100

70

55

40

35

25

15

**S8 Fig.** SDS-PAGE gel of proteins extracted from protein corona of Au NPs after 1 hour incubation in protein rich CCM.
