# Supplementary material for: Analysis of complexes formed by small gold nanoparticles in low concentration in cell culture media
Source: PLoS One. 2019 Jun 14;14(6):e0218211. doi: 10.1371/journal.pone.0218211 (PMC6568402; doi:10.1371/journal.pone.0218211)
Supplement: S1 Table — (DOCX) [file pone.0218211.s009.docx]

**S1 Table**. Identified proteins in the corona

| Band | Protein | Molecular weight [kDa] |
| --- | --- | --- |
| 1 | Alpha-2-macroglobulin | 167 |
| 2 | Inter-alpha-trypsin inhib. Heavy chain | 101 |
| 3 | Lactotransferrin  Kininogen  Gelsolin | 78  69  81 |
| 4 | Serum albumin  Serotransferrin  Alpha-1-antiproteinase  Apolipoprotein E | 69  77  46  36 |
| 5 | IgG heavy chain  Alpha-1-antiproteinase  Alpha-2-HS-glycoprotein  Apolipoprotein E | 38  46  38  36 |
| 6 | Beta-lactoglobulin  Cyclin C  IgG light chain  Fatty acid binding protein  Thioredoxin  Histone H4 | 20  33  12  15  12  11 |
| 7 | IgG light chain  Apolipoprotein AI | 12  30 |
